# Review: Food loss and waste in Sub-Saharan Africa

**DOI:** 10.1016/j.foodpol.2017.03.012

**Published:** 2017-07

**Authors:** Megan Sheahan, Christopher B. Barrett

**Affiliations:** a Precision Agriculture for Development, 32 Atlantic Avenue, Pilot House – Lewis Wharf, Boston, MA 02110, USA; b Cornell SC Johnson College of Business, Cornell University, 301G Warren Hall, Ithaca, NY 14853, USA

**Keywords:** Post-harvest losses, Food loss, Food waste, Sub-Saharan Africa, Review

## Abstract

The research, development practitioner, and donor community has begun to focus on food loss and waste – often referred to as post-harvest losses (PHL) – in Sub-Saharan Africa. This article reviews the current state of the literature on PHL mitigation. First, we identify explicitly the varied objectives underlying efforts to reduce PHL levels. Second, we summarize the estimated magnitudes of losses, evaluate the methodologies used to generate those estimates, and explore the dearth of thoughtful assessment around “optimal” PHL levels. Third, we synthesize and critique the impact evaluation literature around on-farm and off-farm interventions expected to deliver PHL reduction. Fourth, we suggest a suite of other approaches to advancing these same objectives, some of which may prove more cost-effective. Finally, we conclude with a summary of main points.

## 1 Introduction

In September 2015, the United Nations (UN) ambitiously announced a goal of halving worldwide food waste and substantially reducing global food loss by 2030 as part of its Sustainable Development Goals (SDG) agenda. This pledge codifies the huge amount of renewed international attention around reducing the edible losses and waste incurred between farm and fork in the global food system. In Sub-Saharan Africa (SSA), where rural populations depend heavily on food production for their income and food purchases make up a large portion of expenditures in both rural and urban areas, the dialogue is most focused around the theme of “post-harvest losses” (PHL), those that reflect potential consumables that leave farmers’ fields but never make their way into consumers’ mouths. With so much interest and the prospect for significant resource mobilization aimed at reducing PHL, perhaps especially in SSA, it is important to establish what we already know about PHL and interventions aimed at their reduction. Despite plenty of enthusiasm in the development community, holes in critically synthesizing the abundant research still remain.

This review aims to guide development practitioners, donors, and the research community struggling to identify how to devote their time and resources to PHL mitigation. In so doing, we suggest that this community refocus its approach by first returning to the objectives underlying the desire to reduce PHL levels, of which we offer four (Section 2). Then, since most research and interventions to date are merely guided by the estimated magnitude of PHL, we summarize the current state of our understanding of loss levels, critique the methodologies and gaps therein, and explore the lack of thoughtful assessment around the “optimal” PHL levels for which we should be striving (Section 3). We continue by synthesizing the impact evaluation literature around five on-farm PHL reduction interventions, establishing the many gaps that remain in this evidence base, and detailing some of the unique adoption (and dis-adoption) challenges in this arena (Section 4). Because the impact evaluation literature for most existing PHL technologies remains meager and given substantial work specifically on storage technologies, we also discuss four off-farm interventions that may deliver significant PHL reduction alongside broader benefits (also Section 4). We then return to the four objectives we see as guiding the renewed interest in PHL reduction to suggest a suite of other approaches to advancing these same goals, likely with greater cost-effectiveness (Section 5). Finally, we conclude with a summary of main points (Section 6).

We do not attempt to reconcile the competing definitions of food waste, food loss, and PHL or the distinctions between surplus versus loss as proposed by Papargyropoulou et al. (2014). In most work on the topic, food loss refers to anything lost by producers or in distribution while food waste refers to anything lost at the consumer level (de Gorter, 2014), although new work by Bellemare et al. (2017) challenges these existing definitions using a food life cycle approach. These lines are drawn mostly for convenience reasons and are clearly blurred for rural SSA households who often function as both producers and consumers, rendering the categorization essentially meaningless in this context. We prefer to adopt the approach that refers generically to food loss and waste (FLW), a term we use interchangeably with PHL.

## 2 Objectives underpinning investments in reducing PHL

The reasons for the renewed interest in PHL reduction in SSA are best understood by enumerating the multifaceted objectives underpinning the goal recently announced by the UN. One main finding of our review is the lack of a well-defined objective guiding most research and interventions to date, as echoed by the High Level Panel of Experts on Food Security and Nutrition (2014). Most research is motivated only by invocation of the estimated magnitude of PHL and not by what the magnitude means nor by its consequences. Moreover, even when an objective is identified – for example, expanding the supply of grain available to consumers – the full set of subpopulations that might gain or lose from reducing PHL – farmers, middlemen, consumers, etc. – are rarely considered in full. We start by articulating the four objectives that we see as most important in guiding research and interventions on PHL in SSA.

The first objective is ***to improve food security*** via all four internationally recognized pillars: availability, access, utilization, and stability (FAO, 2008). By definition, reducing food loss increases the quantity of food available which can reduce the need to supplement availability through transfer programs (at household level) or via commercial imports or food aid donations (at national level). An increased food supply, under normal circumstances, should also translate into a reduction in prices for consumers, improving overall access. It is no accident that the surge of interest in PHL reduction emerged with the 2007 and 2011 global food price spikes. Retention of inferior quality products, those most likely to be lost currently, could disproportionately benefit the poor where there are price discounts associated with lower quality food (Kadjo et al., 2016). PHL reduction in the form of food quality, for example due to vitamin or protein decay, can improve food utilization (nutrition) among consumers. An increase in retained food can be especially important seasonally in places where the prices of storable staples commonly increase sharply several months after the harvest period, by improving access precisely when seasonally food insecure households most need it and by providing stability.

The second objective is ***to improve food safety***, as distinct from food security. Plenty of food is lost in our system because the quality deteriorates beyond what is acceptable for human consumption. But sometimes spoilage or contamination is not perceptible to the human senses and goes undetected, leading to adverse health effects when food is consumed. Several well-publicized outbreaks of acute aflatoxicosis in SSA – including the death of 125 Kenyans in 2004 – suggest undetected food spoilage with very severe human health implications. Mycotoxins, in the forms of fumonisin and aflatoxins, can lead to slow-developing esophageal and liver cancers (respectively) and are growth-retarding and immunosuppressive even in doses well-short of the more sensational, and often deadly, acute aflatoxicosis. These food safety concerns, arising from fungal or pest infestations, have major disease and global health implications.

The third objective is ***to reduce unnecessary resource use***. These resources come in the form of on-farm inputs that pose sustainability challenges, including water, chemical fertilizer, agro-chemicals, labor, and land. Anticipated PHL by farmers may mean that more of these resources are used than is necessary to meet production or consumption targets. Reducing PHL and, thereby, creating a longer term incentive for farmers to use complementary resources more effectively and efficiently, this line of thinking goes, could ultimately lead to a reduction in the use of scarce resources. Where there may be adverse environmental or human health consequences to use or overuse of inorganic fertilizer (Ayoub, 1999) or pesticides (Sheahan et al., 2017), minimizing unnecessary applications via the reduction of expected PHL could be particularly advantageous. Similar arguments apply to the post-harvest value chain, where reduced PHL could, in principle, reduce fuel costs, transport-related pollution, energy consumption in processing, etc. Not only might limiting input use result in environmental or human health benefits, but it should also reduce costs for farmers, traders, processors, and other actors in food value chains, potentially leading to an increase in profits and a decrease in consumer food prices.

Elaborating on this point, the fourth objective is ***to increase profits for food value chain actors***. The private sector, including smallholder farmers, plays an undeniably central role in making food available to consumers and, at levels above the farmer, establishing a supply chain for producers to utilize. The vast majority of food flows through commercial, not government or non-profit, channels in SSA. Insofar as profit is a natural objective of commercial entities, reducing waste and thereby cost holds natural appeal to private actors in food value chains. In SSA, PHL reduction could mean improving the livelihoods of both smallholder farmers and large-scale agribusinesses. Indeed, recognizing this natural profit motive to reduce PHL that is intrinsic to virtually all actors in food systems in SSA (and elsewhere) is essential to a clear-eyed understanding of the likely benefits to direct interventions that reduce PHL. For the most part, with the partial exception of food safety considerations, private sector actors have a strong material incentive to reduce PHL for their own revenue and profit maximization goals.

## 3 Estimated magnitudes of PHL

PHL can occur anywhere between farmers’ fields and consumers’ plates: at harvest, drying, winnowing, cleaning, on-farm storage, handling, milling, processing, transport, larger-scale mixed storage, retailing, and consumers’ home storage, meal preparation, and consumption. We provide a generalized picture of the post-harvest environment in SSA in the Appendix, detailing where and how losses occur in each of these nodes. It should be acknowledged, however, that the PHL environment varies significantly by crop, country, and even region within country, necessitating a careful review of specific contexts before investing or offering a one-size-fits-all approach.

The urgency of the need to reduce PHL in SSA depends largely on the magnitude of such losses relative to some estimated optimum PHL levels. Similarly, the ability to assess the impact of PHL remediation strategies also hinges on the ability to accurately measure losses before and after an intervention. The sheer number of existing PHL estimates is dizzying, but one of the important takeaways from our review is the lack of consensus on loss size, both in physical mass and value. In this section, we review some of the most recent estimates from SSA, offer explanations for the discrepancies in magnitude estimates, point out the major gaps that remain in our PHL magnitude understanding, and discuss important nuance often overlooked in the establishment of “optimal” loss levels.

### 3.1 Review of estimated magnitudes

Losses and waste can be measured in quantity and quality terms, with important distinctions. Quantity losses occur when the actual amount of food, often measured in either kilograms or calories, reduces over time and space. This is generally the focus on PHL magnitude estimation (and reduction strategies) to date. Quality losses occur via the loss of important nutrients or through contamination of food. These loss types can be more obscure, more difficult to detect, but potentially more important given that micronutrient deficiencies are far more prevalent around the world than is undernourishment due to insufficient dietary energy intake (Barrett and Bevis, 2015) and food-borne health hazards with direct, sometimes catastrophic, effects on human health. When quality losses are sensorily apparent, poor quality may also lead to economic (and thereby quantity) losses when consumers pay a reduced price for inferior quality product (Hodges et al., 2011, Kadjo et al., 2016).

The FAO (2011) estimates that roughly one-third of the physical mass of all food is lost or wasted around the world. In SSA, the estimate is roughly 37 percent or 120–170 kg/year per capita. These estimates are derived from a simplified mass flow model by food group and region using estimates and assumptions from the literature and available macro-level data, such as that published in FAO’s food balance sheets. Lipinski et al. (2013) translate the weight volumes reported by the FAO into calorie terms, reducing the level to a loss of about 23 percent worldwide. World Bank et al. (2011) estimates the value of all grain PHL in SSA to be around USD 4 billion per year. Value and volume estimates like these set off alarm bells for development practitioners and the donor community when global food prices spiked a few years ago and motivate ongoing interest in the topic among both researchers and practitioners.

But there are many methodological reasons to remain skeptical of the FAO and other highly aggregate numbers popularized today. For these reasons, investments have been made in better data collection systems. Most notably in the SSA context, in 2009, coming on the heels of the global food price crisis, the European Commission funded the creation of the African Postharvest Losses Information System (APHLIS), a network of cereal grain experts in eastern and southern Africa charged with accurately estimating PHL for grains across the region. On average across all grains in APHLIS’s current profile, quantity losses range from 14–18 percent between 2003 and 2014 (exclusive of consumer level losses). The FAO (2011) reports a total quantity loss of about 20 percent for all cereals across SSA. The APHLIS numbers, therefore, represent slightly more conservative estimates, either for reasons of methodology, regional specificity, or included crops.

Hodges et al. (2010) and Rembold et al. (2011) describe the database, underlying process, and methodology of APHLIS. In general, estimated losses are pulled from the literature and from data reported by scientists in the region. The value added provided by APHLIS is the interpolation of available data among provinces clustered by climate and crop so as to estimate losses where data are missing. Regional experts alter and update the values over time and space using local knowledge, new data, and year-specific information on production and weather. While a hugely valuable integrative exercise, the main two drawbacks to relying exclusively on this methodology and the loss magnitudes derived from it include (1) the serious oversimplification of a complex PHL environment and (2) the reliance on existing data as an input into their model without being able to scrutinize its quality (Affognon et al., 2015). In other words, APHLIS estimates are only as good as the weakest data input series used.

At the other end of the methodological spectrum one finds PHL estimation methods that rely on highly standardized and carefully supervised household surveys. Kaminski and Christiaensen (2014) estimate the PHL incurred by maize farmers in Malawi, Tanzania, and Uganda using the nationally representative, recently collected, and cross country comparable Living Standards Measurement Study Integrated Surveys on Agriculture (LSMS-ISA). Using responses from farmers to standardized questionnaires, they estimate farmer losses to be only 1.4–5.9 percent of total production. Because these values represent losses on-farm only, they are best compared with the farm-level post-harvest handling and storage loss estimate of 8 percent by the FAO (2011). Household survey responses, therefore, represent a sharp reduction in estimated PHL at that stage in the food supply chain.

Using household survey data as part of the Purdue Improved Crop Storage Project (PICS)^1^ from Benin, Nigeria, Ethiopia, Uganda, and Tanzania, Abdoulaye et al. (2015) also calculate farmer-reported losses of maize, but only specific to the amount lost in storage. Similar to Kaminski and Christiaensen (2014), farmer-reported loss rates are quite low, ranging from 3.7 percent in Uganda to 6.9 percent in Tanzania. While their sample size is large, the underlying surveys were not designed to be nationally representative, precluding their extrapolation to more aggregate levels. But the near match with other estimates derived from nationally representative household survey data points to either an inflation of values from other methodologies or to the fact that farmers do not perceive their losses to be very high, potentially due to the concurrent use of a range of loss-mediating technologies or early sales.

One might reasonably ask whether household survey data are likely to generate reliable estimates of PHL at farm level. The honest answer is that no one really knows. They obviously rely on a farmer’s ability to perfectly recall cumulative losses between harvest and the current point in time and are, therefore, subject to measurement error. But there is little reason to believe that farmers’ measurement error is appreciably greater than that of other sources of loss reporting. The timing of survey implementation may also matter, especially if it does not capture the entire duration of the post-harvest period, so that responses reflect only a fraction of the time crops might be stored. Kaminski and Christiaensen (2014) attempt to correct for this prospective source of bias by estimating full season losses conditional on the timing of questionnaire completion. Likewise, if losses are standard in a community, then farmers might subconsciously report only relative loss rates, thereby underestimating the true PHL magnitude. In the absence of any experimental evidence to compare self-reporting versus directly measured loss rates, it is reasonable to believe that household self-reports based on carefully designed questionnaires implemented using standardized survey protocols to be the best available current data source for farm-level PHL estimates.

The most recent and comprehensive review of work estimating the magnitude of PHL in SSA is Affognon et al. (2015)’s meta-analysis of six SSA countries (Benin, Ghana, Kenya, Malawi, Mozambique, Tanzania) for a variety of crops, not specific to grains. One important and illustrative case is their findings on maize loss. Unlike the common claim that between 40–50 percent of grains are lost in SSA, the literature they summarize yields estimates ranging from 20 percent, where no loss controls are used, to as low as 4 percent, where some loss prevention mechanism is employed. Rosegrant et al. (2015) also estimate the percent of food lost on-farm, up the value chain, by consumers, and in total across SSA by compiling various sources, with findings qualitatively similar to those of Affognon et al. (2015).

Like Rosegrant et al. (2015), many studies attempt to estimate the distribution of losses at particular points along the farm-to-fork chain. The consensus across sources, despite variation in magnitude, is that most grains and cereals are lost during post-harvest handling and storage on-farm, while loss of fresh produce, meat, and seafood is concentrated in processing, packaging, and distribution (Food and Agriculture Organization, 2011, Hodges et al., 2011, World Bank, 2011). In no instance do losses at the consumer level appear significant in SSA (perhaps in many cases because the producer and consumer are the same). As the meta-analysis by Affognon et al. (2015) reveals, over 80 percent of all studies in their sample focused specifically on losses in on-farm storage, so the evidence has been self-reinforcing on this point. Of course, we should expect the distribution to change as supply chains elongate with urbanization and as consumer incomes and the consumption of food away from home grow.

### 3.2 Methodological critiques and gaps

The main takeaway from Affognon et al. (2015) is the inadequacies of loss assessment methodologies across the existing literature – published and unpublished – used to date. Not only is it difficult to compare values due to differences in included crop, levels of the value chain, scale, agro-ecologies, seasonality, and geography, but also the methodologies employed are often unsatisfactory. Methodologies range from modeling/simulation to direct observation to residual methods, all of which have their place when used appropriately, but can be readily misused and misinterpreted. Affognon et al. (2015) call for further research to establish methodologies aimed at accurately measuring losses at levels of the value chain beyond the farm. While most of the existing studies of losses in value chains rely exclusively on case study approaches that are not statistically representative, Minten et al. (2016) pioneered a method of fielding surveys at each level of the value chain to capture wastage figures incrementally, piloting their approach in three countries in Asia. This method offers far more conservative magnitudes of losses than other estimates, underscoring the need to critically examine methodology before relying on estimates and invest in similarly rigorous methods elsewhere, particularly in SSA.

Furthermore, despite a widespread and systematic search of published and unpublished literature, Affognon et al. (2015) – like us – find no conclusive evidence on the magnitude of quality losses stemming from diminished nutritional value or food safety concerns. The only studies we found point to the percent of sampled crops over acceptable mycotoxin levels and in very narrow geographic regions. While most research has focused on mycotoxin contamination in high outbreak areas, Mutiga et al. (2015) find aflatoxin contamination above the regulated limit even in areas with no reports of human fatalities, such as western Kenya. This is likely due to lack of comprehensive surveillance and undetected, sub-clinical human health effects. These findings, among others, raise new concerns about how widespread quality losses, particularly those with the most adverse human health effects, might be in SSA. Other efforts, such as the IFPRI-led Aflacontrol project in Mali and Kenya, aimed to estimate the economic impact of mycotoxins in the SSA food system but was unable “due to a lack of good data” (Wu et al., 2011, p. 9). Estimating the losses derived from degraded nutrient quality may be hugely difficult – especially because food of lower-nutrient quality is likely to still be consumed – but incredibly important to our understanding and ability to address.

### 3.3 Establishing an “optimal” PHL magnitude

The raw estimated magnitude of PHL is often, but inappropriately, used to motivate research and investments in the area. The research and development practitioner community to date lacks a serious assessment of what “optimal” PHL levels may be for meeting their underlying objectives. As de Gorter (2014) lays out cogently, some positive level of PHL is almost surely optimal because eliminating all PHL is prohibitively expensive even with the best imaginable technologies and institutional arrangements. Moreover, because some contamination or spoilage is unavoidable, improving the quality of the food supply sometimes requires incurring some PHL in quantity terms. The appropriate way to frame the question is whether estimated PHL magnitudes exceed optimal PHL rates, but one scarcely, if ever, finds discussions of PHL framed this way.

It is important to understand the microeconomic rationale for PHL before tackling strategies to alleviate the result of those decisions (Barrett, 2015, De Gorter, 2014, Goldsmith et al., 2015, Waterfield and Zilberman, 2012). Some positive level of PHL is economically rational, reflecting a sensible choice by private individuals *not* to invest in loss reduction. There are plenty of instances where waste may be intentional and optimal, at least from the perspective of a private actor whose objective is profit – not *production* – maximization. As but one example, soybean farmers in Brazil willingly increase PHL at harvest (through increases in combine speeds) in order to plant earlier in the first season, make time for a second cropping season, and thereby maximize profits (Goldsmith et al., 2015). This is a poignant reminder that increasing farmer profitability may mean greater levels of food loss, especially where currently available technologies force this tradeoff. Private and social optima may diverge, of course, particularly where externalities are not considered in private decision-making. For whom a given magnitude is optimal is an important question worth exploring when suggesting interventions with a goal of approaching a given PHL magnitude.

Optimal levels, however, may deviate for PHL emanating from quantity and quality reasons. While optimal levels of quantity losses require economic, financial, or accounting-type analysis to establish, removing contaminated and toxic food from the system is crucial and overdue, suggesting that we may need to encourage *more* loss of unsafe food in so much as we know that a potentially substantial amount of food is of problematic quality for human consumption, and even more so for children and infants (Magoha et al., 2014). We offer that in some cases greater PHL levels – that which removes unsafe product from the food supply – can be desirable, while in other cases lower PHL – resulting from less degraded product in the food supply – is the objective. The lack of critical distinction between the magnitude of these losses and the absence of discussion on what should be our targets in each arena suggests the need for more rigor and dialogue in this area.

Another important thread in the discussion of “optimal” is related to the drivers of current loss levels, particularly given evidence derived from household surveys suggesting that farmer-perceived losses are lower than macro-level statistics suggest. Low loss levels do not necessarily imply effective storage or management technologies but may be the result of releasing harvest onto the market early in order to limit *expected losses* due to poor storage facilities. While physical quantity losses, therefore, may appear low, the value and revenue losses associated with quickly depleting stocks, generally when market prices are lowest, may be massive. If farmers were able to confidently store a larger portion of their harvest for longer, revenues may increase with the seasonal increase in selling prices. Kadjo et al. (2015), for example, find that farmers in Benin who believe they will lose more in storage are less likely to hold grain, suggesting revenue losses inversely related to amount stored. By reframing PHL discussions around what is most profit-enhancing for smallholder farmers, related to our fourth objective in Section 2, the underlying “issue” at hand begins to take on a new light.

## 4 PHL mitigation strategies and impacts

To date, significant resources have been leveraged for reducing FLW/PHL around the world. Most interventions in SSA have focused directly on farm-level losses, under the assumption that most losses occur or start on-farm. Few interventions focus on downstream links in the food supply chain, at least not with specific attention to the PHL implications. In the following sub-sections, we review the literature on PHL abatement interventions derived from “modern” or “improved” technologies and any currently available impact analysis.^2^ Impact may come in the form of PHL reduction, farmer or household-level welfare or behavioral impacts (profitability, income levels, food security, savings behavior, nutrient intake, consumption smoothing, expenditure changes, market participation), and higher level general equilibrium impacts (overall food availability and prices that affect individuals beyond just adopters of a new technology). We follow with mention of the major gaps in and challenges for impact analysis approaches to date, thoughts on adoption and dis-adoption patterns, and a review of other off-farm investment opportunities with potentially more broad-based effects.

### 4.1 On-farm interventions

While other technologies aimed at preventing losses may exist or are in experimentation stage, in this sub-section we focus on five PHL reduction techniques used on-farm that have at least some accumulated literature base.^3^ The discussion is largely skewed towards hermetic storage technologies, the PHL reduction strategy that enjoys the most attention in the literature. We focus on effectiveness and impact with respect to staple grains, and refer interested readers to Saran et al. (2012) and Mohapatra et al. (2015) for more on PHL mitigating technologies in horticulture and pulses, respectively.

#### 4.1.1 Improved varietals for pre- and post-harvest loss reduction

Because of the compounding effects of pests and deterioration accumulated before harvest, interventions that aim to reduce PHL while crops are still in the field is arguably more effective than deploying strategies that only start after harvest (Ippolito and Nigro, 2000). John (2014) contends that the focus on PHL exclusively is ill-placed and challenges the research and practitioner community to think more critically about pre-harvest losses as well. One of the most important means of mitigating losses in the field is cultivar selection and development. A major challenge on this front, however, is that hybrid and other higher-yielding varieties are more susceptible to quicker losses post-harvest, suggesting an unfortunate tradeoff for farmers. Indeed, the non-adoption or dis-adoption of yield-enhancing varietals may be a rational choice where the gains are simply lost in storage. Much investment and research is needed to produce high-yielding varieties with long post-harvest lives. Genetics play a major role not only in quantity losses, but also in preventing or reducing susceptibility to myocotoxin contamination.

Affognon et al. (2015) note that very few studies link new varietal development to PHL reduction and impacts, for either quantity or quality reasons, and that those few existing studies only focus on effectiveness in a laboratory setting. Individuals from the PICS project noted that breeding cowpea varieties with longer storage lives was initially unsuccessful, although little is known about the challenges and lessons-learned from this exercise. Otherwise, we are unaware of other interventions in this area. Research and development activities around improved cultivar production that achieves simultaneous goals of increasing productivity, longer shelf life, and reduced likelihood of mycotoxin contamination would be highly advantageous to a multitude of goals.

#### 4.1.2 Education on best practices in harvest and post-harvest handling

Interventions also occur around education for best practices in harvest and post-harvest handling, generally in the form of extension messaging. A manual by Golob (2009) describes the FAO’s approach to extension advice on these two related areas. The most obvious techniques include better sorting: throwing out problematic grains and retaining higher quality for storage. The World Food Programme (WFP) incorporates training on these topics into its multi-faceted PHL reduction strategy with Purchase for Progress (P4P) farmers. USAID invested in post-harvest training of trainer (TOT) activities in SSA related to horticulture, activities described by Kitinoja et al. (2011) as “too short term in nature, too expensive, and too oriented to large-scale commercial horticultural businesses and export crops” (p. 600). Little is known about the tangible impact of these activities as separate from other interventions.

#### 4.1.3 Chemical sprays in storage

It is well-documented, in data from specific regions (e.g., Ngamo et al., 2007) and from nationally-representative household survey data (Kaminski and Christiaensen, 2014), that many farmers use some form of chemical or natural spray during home-based storage as a means of keeping pests and insects away from food. Chemical sprays either take the form of fumigants – applied to deal with already burgeoning infestations – or contact insecticides (or “protectants”) – used to prevent infestations. There are many types of spray to counter particular types of deterioration; the effectiveness of chemical spray use on reducing PHL depends on the type, application techniques, etc. Even when farmers use chemical sprays on stored maize in Benin, Kadjo et al. (2015) do not find that farmers store more maize as a result, suggesting that they do not have full confidence that these sprays decrease losses substantially.

The largest known promotion scheme of chemical protectants in storage is via Malawi’s input subsidy program, which subsidized maize storage chemicals between 2009 and 2012 alongside inorganic fertilizer and improved seed varieties. Ricker-Gilbert and Jones (2015) find farm-household level impacts include an increase in the probability of adopting improved maize varieties and increases in total area and share of area planted to improved maize. The only other known (to us) major international push to increase the use of chemical sprays in storage came from the FAO in the early 1990s. In southern Benin, a new chemical called Sofagrain was promoted alongside use of improved wooden granaries. The socioeconomic impact of this intervention is detailed in Adegbola (2010). The one major welfare impact mentioned in this study is the induced increase of schooling expenditures of adopters.

No more specific welfare impact analysis of chemical spray use appears available. This may be because chemical sprays used in storage (and elsewhere on-farm), particularly in SSA, are known to be of dubious quality (Williamson et al., 2008, Williamson, 2003, Williamson, 2011) and can lead to either short-term (acute) or long-term (accumulating) negative human health effects (Hayes, 1991). Where chemical sprays are used to reduce PHL but inadvertently lead to poor health outcomes, then an unfortunate trade-off exists between PHL reduction and meeting the broader objectives from Section 2. Moreover, indiscriminate chemical application is known to lead to resistant pests (Boyer et al., 2012), making it only a short-term viable strategy before harsher chemicals are necessary. Arthur (1996) explains the expected decline in the use of these “grain protectants” over time. For all of these reasons, promoting more chemical use in storage may not have the greatest positive impact.

#### 4.1.4 Improved storage of grains through hermetic technologies: bags and silos

By far the most widespread intervention strategy, at least judging from the size of the literature, is the use of improved storage devices, especially but not limited to “hermetic” technologies, those that stabilize oxygen levels and offer tight seals to limit the reproduction and life cycle of insects and other pests or pathogens that destroy stored food, especially grains (Murdock et al., 2012). These technologies appear to work well without the coupled application of storage chemicals, another reason for their attractiveness. The two types of hermetic technologies we review are bags and silos (metal or plastic). Various institutions have developed and promoted the use of specialty hermetic technologies, including PICS, the WFP’s P4P, the Effective Grain Storage Project (EGSP), and the FAO. Private-sector led options also exist, including GrainPro, Inc. which offers hermetic and polypropylene bags in addition to other bag and non-bag storage options for commercial purchase.

A recent (2014) special issue of the *Journal of Stored Products Research* was dedicated to synthesizing the available research on PICS, one of the most heavily funded and widely celebrated interventions in on-farm storage improvements for mitigating PHL. Most of the issue focuses on the ability of the bags to reduce losses relative to baseline levels. The effectiveness of hermetic bags for storing maize in Kenya was also studied by De Groote et al. (2013) and cowpea in Niger by Baoua et al. (2012), both of whom also find considerable loss prevention, like many others. The main critique of this literature, as described in more detail by Affognon et al. (2015), is that many – although not all – of these studies draw conclusions about the effectiveness of their hermetic technologies based on controlled laboratory settings, not actual and often imperfect use by farmers under their varied constraints and operating environments. The WFP (2014) conducted its own study of the effectiveness of its pilot PHL interventions using farmer responses. Their analysis, which reported that farmers retained 98 percent of their harvest via these new methods over 100 days, was not specific to hermetic bags or silo use, but rather reflects the suite of PHL interventions they provided farmers, including extension services for harvesting, handling, drying, threshing, etc. We are unaware of any external evidence from independent researchers on the effectiveness of the intervention or a careful breakdown by technology employed.

It is not enough to show that these hermetic technologies work to prevent losses; the investment must also imply higher profits for farmers or others undertaking storage. Jones et al. (2014) show that, on average, PICS bags offer higher levels of profitability for farmers than government-subsidized chemical storage protectants in Malawi. Kimenju and De Groote (2010) study the economics of the “super” GrainPro bag in Kenya and find that these bags were not economically attractive because the loss abatement was not substantial enough to make the necessary annual purchase profitable. Ndegwa et al., 2015a, Ndegwa et al., 2015b use randomized control trial (RCT) methods to study the profitability of hermetic bag technology in Kenya, bags that do not need to be replaced repeatedly, estimating a benefit-cost ratio of 1.6 for one season and 4.8 for three seasons, signaling a sound investment for farmers. Kimenju and De Groote (2010) find that the returns to metal silos are high and increase with silo size, but only in the long run after benefits accrue.

But to what extent have hermetic bags or silos helped to achieve any or all of the underlying objectives of PHL reduction, as enumerated in Section 2? Only two rigorous impact studies exist (to our knowledge). Gitonga et al. (2013) use propensity score matching (PSM) techniques to analyze the difference in adopters and non-adopters of metal silos in Kenya, finding that adopters experience almost the complete elimination of losses due to insects, an increase in 150–198 kg/household of available maize grain, a reduction in expenditures on chemical storage sprays, an increase in home-based maize consumption by 1.8–2.4 months (a decrease in market reliance), an increase in wait time before selling grains on market (higher prices received for sales), and a reduction of time associated with food insecurity by one month. On the contrary, Cunguara and Darnhofer (2011) find no significant impact on household income levels in nationally-representative data from rural Mozambique through the adoption and use of improved granaries, although provide no details on the specific types of granaries under investigation.

There are no similarly rigorous impact analysis studies for hermetic bags, to our knowledge. The WFP (2014) report makes big claims about increased food security and other impact measures, but provides no actual data or description of methodology used to arrive at these conclusions. We understand from private communication that welfare analysis is planned on the PICS project, but only after another round of data is collected. We are also unaware of any evidence linking the use of hermetic or any other improved storage option with mycotoxin levels or other food quality outcomes, including nutrition.^4^

#### 4.1.5 Integrated pest management in storage (IPM)

The integrated pest management (IPM) paradigm is generally discussed with respect to the prevalence of pests pre-harvest, but IPM can also be useful during storage. With the outbreak of grain borers, in particular, and given concerns about indiscriminant use of potentially toxic chemicals in storage, biological control – the release of natural biological predators into a system to control unwanted and destructive pests – has been suggested as an alternative. Indeed post-harvest IPM control may be easier and more effective than pre-harvest IPM control due to the enclosed nature of storage devices within which biological control agents would be released. For a more extensive overview of how IPM works in storage, with a developed country perspective, see Scholler et al. (1997).

The economics literature has not considered the effectiveness, profitability, and impact dimensions of IPM in storage. The only known paper to model any element of farmer behavior linked to IPM adoption in storage is Waterfield and Zilberman (2012), with a focus on optimal pest management decisions from both private and social perspectives. Adda et al. (2002) study an IPM experiment conducted on stored maize in Togo by the International Institute of Tropical Agriculture (IITA). These researchers found the IPM-based storage methods, as trialed on smallholder farms under normal conditions, were just as effective as chemical-based methods in reducing grain infestation and losses and significantly better than longstanding and rudimentary storage methods. The IPM strategy also involved grain sorting before storage, linking back to pre-storage handling interventions, so as to dispose of potentially problematic grains before larger contamination could take hold.

Haines (2000) discusses the many challenges to a more robust use of IPM for storage in developing countries, not the least of which is the lack of research to help better advance the technologies and adoption. Until physical and social scientists are able to tackle these known challenges, IPM will likely remain a marginal contributor to PHL reduction strategies.

### 4.2 Impact evaluation critiques and gaps

Our review demonstrates that the literature offering rigorous estimates of the on-farm impacts of PHL reduction technologies is thin at best. The evidence that exists largely relates to changes in physical losses. There is negligible systematic evidence of quality premiums paid to staple crop producers, which might help encourage investment in PHL remediation and quality control processes. Very little is known about actual welfare impacts at the household level on a range of indicators – income levels, food security, savings behavior, consumption smoothing, expenditure patterns, investments, market participation, etc. – apart from the few studies highlighted. There is also a serious lack of linkages with mycotoxin contamination levels or other food quality or safety metrics, including nutrition indicators.

As best as we can tell, nothing is known about general equilibrium impacts of PHL reduction efforts specifically, i.e., of the ways in which on-farm PHL interventions affect non-adopting farm households or actors further down the value chain, including consumers. Many questions remain. For example, how do even modest rates of on-farm PHL technology adoption affect local level market supply conditions, especially as related to seasonal availability? Do these potential changes in local supply affect prices? Are they able to stabilize prices interseasonally, reducing the magnitude of the difference between low post-harvest and high lean season prices? Do on-farm PHL interventions simply displace losses until further down the value chain, especially when other intermediaries do not have the capacity to absorb the supply? How do greater levels of food supply affect incentives and behavior of transporters, wholesalers, retailers, etc.? Tomlins et al. (2007) expose that sweet potato traders in Tanzania are not interested in buying/selling stored roots. Do preferences like these create disincentives for farmers to want to invest in on-farm PHL reduction? Is there a knowledge gap at higher levels of the supply chain that will offset any loss remediation on-farm? In the end, do the abated losses actually reach consumers?

Femenia (2015) studies the world-wide implications of public storage subsidies in an industrialized country context and finds that these policies can have destabilizing effects on world agricultural markets. While not entirely relevant in the SSA context, the study does provide a useful caution about the broader range of unintended or unaccounted for impacts of PHL reduction strategies. Tracing the impacts of one intervention through the supply chain is an incredibly difficult task, especially when pursuing causal impacts, but an important one if we are to understand how PHL investments stack up against the underlying objectives from Section 2.

### 4.3 Adoption barriers and dis-adoption patterns

It is difficult to pull much from the small literature on adoption patterns since most underlying data sets are not representative of any meaningful population. Affognon et al. (2015) finds that studies of adoption levels show percentages ranging from 12 to 74 across technologies, crops, and countries covered. They only note one study where dis-adoption was recorded: 56–73 percent of initial adopters in the study on Sofagrain and improved granary structures in southern Benin (Adegbola, 2010).

Many studies delve into the drivers of adoption, offering similar stories to barriers found in other parts of the agricultural technology adoption literature: income and credit access constraints, education or other knowledge barriers, gender-sensitivity issues, labor availability bottlenecks, etc. In their impact analysis study, Gitonga et al. (2013) found major differences in socio-economic and other baseline characteristics across adopters and non-adopters of metal silos in Kenya, signaling that these technologies are still only within reach of the relatively more affluent or productive farming households.

Other studies point to adoption constraints that are more specific to these technologies. For example, Kadjo et al. (2015) study the determinants of storage decisions among maize farmers in Benin. In their sample, farmers make decisions about PHL technology use based on their expectations about loss size; those that expect higher losses are more likely to release their grains onto the market more quickly and not use an improved storage technique. Matita and Dambolachepa (2015) study adoption of post-harvest management technologies in Malawi over the period where chemical insecticides used in storage were subsidized by the government. In addition to other adoption challenges, they also found the fear of theft was a major deterrent to farmers using improved storage options external to the household dwelling.

Low adoption rates of on-farm PHL prevention may also be an expression of low expected returns to investment in potentially very useful storage devices. While several papers found favorable benefit-cost ratios for PHL interventions, particularly hermetic storage, these profitability dimensions may not be internalized by farmers. One of the findings from the various benefit-cost calculations is the known level of production and swings in prices after harvest necessary for a given storage device and size to be profitable. These conditions should be explained to farmers fully. For other technologies, only the efficacy of the PHL intervention is known (and sometimes only in a controlled laboratory setting), not the profitability dimensions given actual resource-constrained farmer conditions.

Affognon et al. (2015) provides several instances of well-intentioned technologies not performing optimally when used on-farmers’ fields due to the many constraints not considered or accounted for in design or failures in extension messaging on proper use. It is well-noted that farmers have many questions about these technologies, some of which extension agents are not equipped to answer. One of the most costly components of some interventions is the training and extension programming included to help farmers understand how to use the technologies effectively; a significant part of the PICS project is devoted to providing village-level demonstrations. Baoua et al. (2013) list and answer the set of questions they most commonly heard from farmers using or considering hermetic bags in rural Niger. Baributsa et al. (2010) offer innovative suggestions on how to communicate knowledge about best practices surrounding hermetic storage by coupling purchase with cell phone-based extension outreach.

### 4.4 Off-farm interventions with potentially more broad-based benefits

In their meta-analysis, Affognon et al. (2015) found that only 19 percent of the papers they surveyed mentioned a technology for mitigating PHL beyond the farm-level. And within this small set of papers, they often focused on non-staple grain foods, like highly perishable fish or fresh fruits and vegetables. But, given we know so little about the true and diffuse impacts of PHL reduction strategies on-farm, it may also be appropriate to consider off-farm interventions with more broad-based benefits. The type of investments that we survey in this sub-section may not be specifically under the guise of PHL reduction, but benefits are expected to accrue in PHL reduction given the interconnected nature of food systems and the endogeneity of PHL rates to food system agents’ behaviors. Several of the strategies mentioned below also appear in Hodges et al. (2011).

#### 4.4.1 Infrastructure improvements

Investments in food marketing infrastructure (roads, market facilities, electricity, etc.) are known to induce behavior changes along agricultural supply chains, with potentially major direct reductions in PHL as well as indirect reductions via complementary private investments. For instance, Minten et al. (2014) study the rapid rise in the number and capacity of cold storage facilities in a potato-producing region in India. Through key informant interviews, they find that the private sector was willing to make major investments following policy changes and investments made by the government, including widespread infrastructure improvements.

Rosegrant et al. (2015) econometrically test the relationship between investment across a suite of infrastructure variables and PHL levels, finding that the following are the largest contributors to PHL reduction: more extensive paved road networks, higher usage of railways, and higher consumption of electricity. They also estimate the average size of investment necessary and the food security implications through a reduction in global prices. An invited response to these findings suggests that the estimated benefits to food security that occur through PHL reduction directly may be exaggerated because some of the decrease may come indirectly through other benefits to infrastructure advances, such as increased uptake of improved cultivars, reduced transport costs that affect prices, etc. (Barrett, 2015).

#### 4.4.2 Warehouse receipt systems

Another avenue for simultaneously reducing PHL while tending to other important rural objectives in SSA is investment in warehouse receipt systems (WRS). Coulter and Onumah (2002) explain the role of WRS in the region. The quick removal of food from smallholder farms to more modern and well-regulated centralized storage areas can decrease storage losses significantly, especially when using improved shelf-life technologies (e.g., first-expired-first-out) as described in Maarten et al. (2014). We, however, find no specific evidence linking WRS to PHL abatement in SSA.

WRSs work to “facilitate market exchange by reducing transaction costs and imperfect information [that] will benefit the agricultural trade in Africa” (Coulter and Onumah, 2002, p. 322) and may also help to improve the service of SSA’s commodity exchange systems, several of which are currently under-performing (Sitko and Jayne, 2012). WRSs can serve as a means of collateralizing producer credit, addressing financial market failures that commonly discourage storage by liquidity-constrained farmers (Stephens and Barrett, 2011, Burke, 2014). Like infrastructure improvements, the many indirect benefits of WRSs could also help to tackle the objectives underlying PHL reduction work.

#### 4.4.3 Rural financial market development

Given the challenges to adoption of established PHL technologies, in some part related to income and credit constraints, one intervention with both direct and indirect pathways to PHL reduction, as well as to the objectives that motivate PHL reduction, is rural financial market development. When they work effectively, financial markets provide smallholders with access to credit, savings, and insurance that help facilitate investments in PHL remediation. Kadjo et al. (2015) suggest pairing access to credit with access to hermetic storage in order to promote increased farmer uptake alongside other benefits. Implicitly, this recognizes that farmers who cannot readily access financial services may currently store their grains as a form of in-kind savings, or sell their grains only to predictably buy back later in the season as a form of in-kind credit (Stephens and Barrett, 2011, Burke, 2014).

Farmer choices with respect to storage and storage technologies are thus intimately bound up with farmers’ access to modern financial services. A significant development economics literature details the many ways in which financial market failures manifest in seemingly-inefficient behaviors in commodity markets, technology uptake, etc., but that the best approach to addressing these ‘displaced distortions’ is commonly to address the root underlying financial market failure rather than to treat the behavior – such as inefficient storage with a high PHL rate – that is merely its symptom (Barrett, 2007). Credit, insurance, and other financial product market development are widely expected to trigger household and firm investment in a larger suite of on-farm and post-harvest technologies and perhaps in collective marketing strategies that help to tackle some of the underlying objectives that motivate PHL reduction.

#### 4.4.4 More efficient food value chains

One of the major developments of the past two decades in developing country agriculture has been the rapid rise of modern food value chains (Reardon et al., 2003, Reardon et al., 2009, Gómez et al., 2011). Vertical integration and expanded contracting from food retailers, wholesalers, and restaurants has typically led to significant improvements in logistics broadly, including reduced PHL rates. A potentially interesting option related to transport is the use of computer-based modeling and monitoring systems that optimize and adapt the route and scheduling of transport via a method piloted by Venus et al. (2013) for the movement of tomatoes between producing regions in Burkina Faso and consuming markets in Ghana. In other regions of the world, researchers note the role of uneven power dynamics between actors in value chains in determining the size of PHL, mainly due to the inability to transmit information that could be potentially useful to all parties (Xhoxhi et al., 2014). Funding innovative methods of lessening information constraints and bottlenecks within the rapidly evolving value chain may serve as a way to create more efficient markets with the added benefit of reducing PHL.

## 5 Alternative approaches to achieve the same objectives

de Gorter (2014) makes the case that while simple arithmetic implies that reducing PHL would feed more people – helping to tackle the first objective described in Section 2 – the cost of doing so is hugely prohibitive, suggesting that other approaches to achieving the same objectives are worth exploring. In this section, we consider some (of many) alternate approaches to achieving the four noble and important objectives enumerated in Section 2. Rosegrant et al. (2015) and Barrett (2015) both argue that while PHL reduction investments might pass the most basic benefit/cost > 1 test, and in some cases significantly greater than one (e.g., Ndegwa et al., 2015a, Ndegwa et al., 2015b), multiple other candidate interventions seem to dominate PHL investments in terms of delivering on one or more of the objectives used to motivate PHL remediation strategies. We do not concern ourselves with ranking the benefit/cost ratios but rather suggest these other and likely more effective means of achieving the same objectives.

### 5.1 Objective 1: improve food security

Barrett (2015) stresses that poverty, not PHL levels, is the main driver of food insecurity. And no one seriously argues that PHL is a significant driver of poverty. It follows that other ways of reducing poverty may offer more direct, lower cost means of advancing food security objectives than PHL reduction investments. We note the more fundamental importance of poverty reduction to improving food security because as incomes inevitably rise, PHL volumes may *increase* for various reasons, including (i) as food appears cheaper relative to income, households may over-purchase in order to avoid stock-outs, resulting in greater PHL, and (ii) as diets diversify towards more perishable meat and fruits and vegetables, higher PHL is a natural result of shifting composition of consumers’ food basket. PHL and food insecurity rates do not necessarily move in the same direction.

In order to reduce poverty, we must identify strategies that raise income levels of the poor. Because the bulk of today’s poor in SSA still rely on agriculture-related livelihoods, a focus on poverty alleviation will necessitate agricultural productivity growth in most parts of SSA, returning emphasis to pre-harvest interventions that bolster productivity growth, especially labor productivity growth that is the main driver of poverty reduction (Christiaensen and Demery, 2007). Focusing on pre-harvest losses in SSA is critically important given the size of the “yield gap” relative to nearly all other regions of the world. This approach may involve reducing widespread uncertainties in agricultural production through the use of better varietals, on-field pest control, and crop disease prevention (de Gorter, 2014). It may also involve the continued promotion of appropriate modern agricultural inputs beyond what is already being used on-farmers’ fields (Sheahan and Barrett, 2017).

### 5.2 Objective 2: improve food safety

Mutiga et al. (2015), among others, suggest that fumonisin, aflatoxin, and other mycotoxins contamination are more widespread than previously appreciated. Instead of focusing purely on PHL reduction, a potentially more impactful approach might involve investing in greater food safety surveillance. Mutiga et al. (2015) advocate that local grain mills may be the most appropriate venue for the detection of mycotoxins in local food supplies since rural households often bring their grains to mills for grinding. Testing is necessary for many mycotoxins because visible inspection only identifies severe cases and quite imperfectly. Investing in low-cost testing and analysis is suggested by Wagacha and Muthomi (2008). Harvey et al. (2013) offer a review of simple diagnostic tests for aflatoxin specifically. Again, detecting and removing unsafe food from the system before it reaches consumers may actually *increase* PHL levels if it leads to disposal or destruction of unsafe commodities.

Investments in food safety in SSA may consider established approaches within food value chains globally, such as the systemic Hazard Analysis Critical Control Point (HACCP) process mandatory for many food sub-sectors in high income countries (Pierson, 1992, Mortimore and Wallace, 2013). Systematic processes to detect biological, chemical, and physical contaminants become essential to ensuring food safety, especially for food that travels through the hands of many intermediaries, which is increasingly the case as SSA food value chains elongate with urbanization. FAO has actively promoted HACCP and similar food quality assurance systems and procedures in the marketing of fish in developing countries, but these have not extended across sub-sectors or countries. A further benefit of investment in food safety comes from signaling higher product standards which may lead to increased opportunities for exports and access into international markets for SSA producers and processors (Swinnen et al., 2015).

### 5.3 Objective 3: reduce wasted resources

Reducing PHL towards the objective of reducing wasted resources and expenditures, especially on-farm, can also be achieved through agricultural productivity growth and efficiency gains. A significant share of innovations aimed at agricultural production and processing promote cost-reducing gains through more efficient conversion of inputs into product. Particularly given evidence of low levels of paired inputs with known complementarity gains in SSA farming (Sheahan and Barrett, 2017), reallocation of resources already on-farm could lead to productivity gains without the use of additional inputs. There is no evidence that PHL remediation outperforms more conventional technological change or extension efforts to improve the efficient use of new technologies in terms of resource conservation. Investments in this area could also involve simple soil testing to the aim of only applying the fertilizers that will be taken up by plants and result in yield gains.

One could also envision several investments that seek to reduce the “resources” embodied in the wasted food instead of the food itself. This might involve creating or bolstering secondary markets for imperfectly appearing edible food (de Gorter, 2014); differentiating products and creating parallel supply chains for new or alternative products also suggest growing profits along the value chain (see objective 4). It could also involve diverting inedible food towards safe composting or energy-creation, for example, by turning aflatoxin contamination grains and groundnuts into charcoal briquettes (Filbert and Brown, 2012). At the same time, it should also be noted that currently “lost” food left on-farm may deliver eco-system services benefits, including delivering nutrients to the soil or carbon sequestration.

### 5.4 Objective 4: increase profit for food industry firms and farmers

Traditional food value chains in developing countries, as defined by Gómez and Ricketts (2013), exhibit the highest PHL rates but also deliver nutrients to consumers most cheaply. As value chains morph with the rise of a middle class and changing preferences, PHL remediation strategies will be forced to shift from a focus on PHL alleviation on-farm towards more downstream levels of the value chain (Affognon et al., 2015). Minten et al. (2012) observe exactly this in private investment in cold storage in Indian crop value chains. The task then will be to create an environment that induces substantial private investments as opposed to undertaking those investments directly. Such investments include the physical and institutional infrastructure on which private intermediaries depend, as well as other public goods and services such as research and extension throughout the value chain, police protection, contract enforcement and dispute resolution services, etc.

## 6 Conclusions

The resurgence of activity and interest in reducing post-harvest losses in Sub-Saharan Africa – largely thought of as the counter-strategy to increasing productivity – encourages a thoughtful synthesis and critical review of the available literature. This article helps to establish several important points about the current state of our understanding and reveals the even larger set of unanswered questions that will help to guide more appropriate research and investments. We summarize nine key points:

1. There exist four distinct, underlying objectives of PHL reduction work that most interest the development community: (1) improve food security, (2) improve food safety, (4) reduce wasted resources, and (4) increase profits along the food supply chain.
2. There remains lack of consensus on the estimated magnitude of PHL in SSA, and even the “best guess” estimates are problematic for a variety of reasons. Furthermore, little reliable information about losses incurred beyond the farm level is currently available. We highlight a new survey method developed and employed in Asia as one for potential replication in SSA.
3. Food quality losses, as separate from food quantity losses, are likely more widespread in SSA than commonly acknowledged. Unlike efforts to better establish the magnitude of and find innovative ways to curtail or detect quantity losses, we find no similar effort related to the pervasiveness of food of substandard quality in SSA.
4. The development community lacks a serious assessment of what “optimal” PHL levels are for meeting any of the four underlying objectives identified in point 1. Given that remediation is costly, optimal PHL rates are surely positive. Yet much of the discourse and literature seems to assume that zero PHL is both feasible and optimal. Optimal levels of PHL may diverge, however, for PHL emanating from quantity versus quality/safety reasons, especially conditional on current technologies.
5. Current estimates of the magnitudes and distribution of losses suggests that most PHL in SSA occur on-farm. As a result, most interventions aimed at reducing PHL have also been directed on-farm, almost entirely around hermetic storage (bags and silos). There is a swell of literature about these products and their efficacy in reducing PHL, although more often than not from controlled settings. There are few other evaluations of innovations related to on- or beyond-farm technologies.
6. Despite many organizations working on improved storage options, there is a very thin literature on the human welfare impacts or on the impacts with respect to any of the other underlying objectives of PHL (from point 1) related to these technologies. We identify only two studies that employ rigorous evaluation methods that uncover interesting (although contradictory) household level impacts. The literature is also mostly silent on the food quality and safety impacts of these technologies.
7. Little is known about broader, general equilibrium impacts, the ways in which PHL interventions affect non-adopting farm households as well as actors further up or down the value chain, including consumers. We offer a range of important research questions that have yet to be answered, those most important for understanding the cumulative impact of PHL reduction strategies and the distributional consequences among different sub-populations.
8. Because the impact evaluation evidence for most existing PHL technologies remains meager and given a crowd of organizations working on storage issues, there may be good reason to consider off-farm interventions that deliver PHL reduction alongside other more broad-based benefits. While other strategies exist, we highlight four: (1) infrastructure improvements, (2) warehouse receipt systems, (3) rural financial market development, and (4) more efficient markets and value chains.
9. The cost of PHL reduction is likely massive, and the full benefit/cost of many PHL interventions remains largely unknown. The limited, imperfect efforts made to date to estimate relative benefit/cost suggest that PHL reduction is *not* the most cost-effective means of achieving our objectives (from point 1). We offer a limited set of non-PHL approaches to tackling these same goals.

The many unanswered questions emanating from these points suggest that investing in a better understanding is crucial before funneling more funds and attention to PHL reduction technologies or processes that may have little payoff. Indeed, the most important place to start might be asking a series of even more foundational questions: if PHL rates appear high, why? Is that perhaps a conditional optimum or do we think agents are systematically making mistakes? If they are making mistakes, is it due to information gaps that can be remedied? Or is the problem that the conditional optimum is constrained by insufficient technological options that can be remedied through product innovation or diffusion? Or is a seemingly high PHL rate merely a symptom of broader systemic problems – e.g., rural financial market failures, poor institutional and physical marketing infrastructure – that merit attention irrespective of PHL considerations? Or is a high PHL rate the byproduct of a highly efficient system that results in food cheap enough that people can afford to lose some?

Any researcher or organization committed to advancing the four objectives we outline needs to pay attention to PHL in SSA given widespread concerns that such rates are, in some vague sense, “too high.” Yet there is a striking absence of evidence that the rapid increase in attention to and investments in PHL remediation for SSA is paying significant dividends. In part, we suspect this has to do with the remarkable lack of attention to date in attempting to identify what optimal PHL rates might be for different actors in food value chains, given the broader array of (often non-agricultural) constraints and incentives they face, and thus whether PHL remediation efforts are likely to induce uptake and impact. The lack of impact evaluation evidence likely results from a near-exclusive focus on reducing PHL quantities and insufficient emphasis on food safety and quality assurance. A narrow emphasis on reducing food volumes lost post-harvest might even inadvertently aggravate and already serious issue of high rates of food contamination in SSA. With so many important gaps in current knowledge, more emphasis needs to be placed on coordinated learning, especially assessment of whether PHL remediation investments are relatively cost-effective in advancing the four core objectives that motivate such initiatives: improved food security, food safety, and profitability, as well as reduced resource use.

## Footnotes

1 The PICS program was originally calls the Purdue Improved Cowpea Storage Program, but the mandate has since expanded to other crops.

2 We do not study traditional approaches via botanicals, vegetable oils, dusts, solar, and underground pit storage.

3 For example, one idea in experimental stage is the use of acoustics to detect pest outbreaks in storage. Kiobia (2015) provides the engineering details on design and functionality, with specific consideration of the constraints faced by maize farmers in Tanzania. Little is known about the viability, profitability, and impacts of this technology, but the fact that this research exists suggests that further innovations for PHL reduction are currently underway.

4 Another complementary goal of promoting hermetic bag and silo use is the opportunity to simultaneously develop a local supply chain for the production and distribution of these products. We refer interested readers to Gitonga et al., 2013, Ndegwa et al., 2015a, Brennan and Gooding, 2015 for more on this topic.
