# Supplementary material for: Review: Food loss and waste in Sub-Saharan Africa
Source: Food Policy. 2017 Jul;70:1–12. doi: 10.1016/j.foodpol.2017.03.012 (PMC5555439; doi:10.1016/j.foodpol.2017.03.012)
Supplement: Supplementary data 1 [file mmc1.docx]

**Appendix**

In this appendix, we provide a short overview of where and how losses – both in quantity and quality terms – occur between farm and fork in SSA.

*Pre-harvest and harvest*

Pre-harvest, farmers in SSA fully expect some element of loss before harvest on account of pests, rodents, weeds, poor or erratic rainfall, storm damage, flooding, and sub-optimal management. At harvest time, low labor availability, time constraints, or lack of adequate storage or market opportunities may make it economically rational for farmers to leave some of their crop in their fields, unharvested. Imperfect human handling of crops at harvest, widespread in traditional SSA agriculture, often results in on-site losses, but also transfers oils from human hands to crops which may result in deterioration later in storage. Further, pre-harvest management decisions (including cultivar selection and pest management while in the field) significantly influence PHL outcomes (e.g., Ippolito and Nigro 2000). These instances reflect losses in quantity even before post-harvest processes kick in.

Quality losses are just as important as quantity losses prior to and during harvest. Crops can be contaminated with naturally-occurring toxic fungi during cultivation due to stress conditions (from drought, pests, soil degradation, etc.), management decisions (e.g., planting date), genetic factors of crops, and the preferences of local pathogens (Hell *et al.* 2008; Wiatrak *et al.* 2005). The continuing degradation of soils, including through depletion of minerals essential for human health, represents another avenue for pre-harvest quality loss, especially important since most of the minerals ingested by humans come from either soil or water (Allaway 1986).

*Drying, winnowing, cleaning*

After harvest, many grains and some vegetables may require or benefit from drying prior to consumption or storage. Heavy reliance on sun drying techniques, with exposure to elements, is one important on-farm space where rapid biodeterioration starts. While all crops deteriorate, the pace of deterioration can be altered by maintaining an environment conducive to long “shelf” life. Climate change introduces more variability and less predictability in weather and moisture patterns, potentially exacerbating the difficulty of properly drying grains without machinery (Stathers, Lamboll, Mvumi 2013). Quality losses in the form of nutrient degradation can occur during drying, as well. The technique used to dry orange-fleshed sweet potatoes on-farm, for example, is highly related to the amount of carotenoids retained (Bechoff *et al.* 2011). Some grain crops, including millets and sorghum, also require threshing while others, like maize and rice, may require winnowing or cleaning before storage. Manual and rudimentary processes, also widespread throughout SSA, commonly lead to a higher loss of grain than do mechanized methods.

*On-farm storage*

Most farmers store agricultural products for at least some time before consuming at home or selling at market. Suboptimal pre-storage drying practices are common preconditions for the accumulation of mycotoxins during storage (Hodges *et al.* 2011).Traditional storage shelters made out of natural materials often harbor or fail to keep out produce-eating pests and/or are unable to protect food from humidity, temperature variability, etc. that result in the vitamin or protein breakdown or food spoilage. Traditional storage methods, including botanicals and ash, in addition to the use of insecticides and fumigants are often used in storage to keep infestations to a minimum. Even where storage structures are modern, loss at this juncture may be stochastic or unavoidable for a host of other reasons, including electricity interruptions, storm damage, etc.

*Handling, milling, and processing*

Between storage and consumption, several other post-harvest processes may occur that result in losses. Over the past three decades, there has been a significant move from home-based milling of grains (through laborious pounding) to small-scale milling operations throughout SSA (World Bank *et al.* 2011). It remains unknown how this new institutional arrangement has changed the magnitude of quantity loss at this stage. Rough handling and processing is known to be responsible for losses of fresh fruits and vegetables, in part due to loss of aesthetic appeal to consumers.

Milling and processing commonly leads to quality degradation, especially in the form micronutrient loss (Welch 2001; Barrett and Bevis 2015). For example, modern polishing of rice removes bran and germ of the grain that contain much of the iron, zinc, calcium, vitamins, phytate, and some of the protein in rice (Lauren *et al.* 2001). As with rice, modern milling of wheat typically removes both the bran and the germ of the wheat grain, sheering away many of the vitamins as well as most of the minerals and healthy oils carried in wheat grain (Welch 2001; Pollan 2013). Welch and Graham (1999) show that rice and wheat lose 69 and 67 percent of their iron contents to milling, respectively, as well as 39 and 73 percent of their respective zinc contents. Processed cereals of all types have lower levels of minerals and vitamins in the absence of a concerted (and costly) effort to fortify the food later.

*Transport*

With the lowest road network densities in the world (World Bank 2009) and inadequately surfaced roads where they exist, poor transportation infrastructure throughout SSA naturally results in PHL for various reasons. First, poor rural market development, high cost of transport, few traders, and other marketing intermediaries operating in rural areas may mean that grains sit in storage longer than necessary, repeating or exacerbating the storage losses described above. Second, because intermediate storage facilities often do not exist in rural areas, food may need to be transported farther than necessary on first haul in order to sell before spoilage, increasing overall transport costs (Barrett 1996). Third, even when food does move between the farm-gate and market or higher order storage facilities, the lack of cold storage or climate control during transport as a result of poor technology and logistics leads to further PHL. Sub-optimal humidity conditions may encourage spoilage and other food quality loss where mold or fungus takes hold. Fourth, suboptimal road surface condition and packaging can also mean that food is damaged or lost during transport, particularly when not kept in containers that safeguard losses. Finally, because transportation costs are high, vehicles are often over-packed to limit redundant travel which compresses (and possibly ruins) food and can lead to higher accident rates, which exacerbate PHL.

*Large-scale/mixed storage*

Food may also sit in larger-scale and mixed storage facilities for some time before consumption, either at private silos or warehouses or in government or multinational agency depots. Several SSA governments maintain heavy control over grain reserves in large-scale storage facilities. While facilities are generally far more modern than on-farm structures, losses appear high for a range of other reasons, including corruption, indiscriminate applications of insecticides and pesticides (leading to resistance and increased pest pressure over time), as well as poor timing/planning on when to release grain reserves into the market. For example, Zambia’s Food Reserve Agency, a government-run parastatal food reserve and maize marketing board, sometimes struggles to balance pledges made to farmers about the amount of maize they will buy and the excessive amount they hold in storage (Mason and Myers 2013). In the 2012/13 season, it is estimated that 32 percent of the reserves were lost in government storage as a result of excessive volumes and the inability to release onto the market without causing significant price distortions (Sitko and Kuteya 2013). Similar stories, related to policy choices and food security strategies on the part of numerous SSA governments, exist elsewhere in the region.

*Retailing*

PHL incurred during retailing are often related to overstocking food in piles to create attractive displays for consumers, a problem more likely in developed country contexts than in SSA (Hodges *et al.* 2011). Losses also occur at the retail level when backstream storage facilities are inadequate, forcing food to reach retail in volumes mismatched with consumer demand. Retailers may also dispose of food that does not have the appearance of being high quality in order for their selection to be more attractive (e.g., blemish free, appropriately sized, not misshapen) to discerning buyers or those concerned about the safety/quality of their purchases. Finally, if they face a significant consumer penalty from stock-outs, retailers and restaurants may optimally overstock many perishables, causing an increase in PHL. Since food-away-from-home is the food expenditure category with the highest income elasticity of demand and the most rapid growth due to urbanization, economic growth and demographic change in SSA will inexorably lead to expansion in this element of PHL. In urban areas and growing towns, the inevitable increase in PHL at this level may create compounding issues for waste management systems.

*Consumers*

After consumers procure food from markets, PHL occurs within the household, often labelled “consumer waste.” This happens where refrigeration and other food storage capacities are low, when consumers are unable or unwilling to smooth purchases over time and need to buy in significant bulk, etc. The rapidly changing nature of food consumption behaviors due to increases in incomes and urbanization typically lead to higher rates of consumer-level waste for the same reasons it does at retail level (Kearney 2010). These trends may also lead more individuals to purchase more food for home use than necessary, with full knowledge that some of it will be lost, purely as a matter of convenience. Rising incomes also imply shifting preference towards higher quality foods that demand more sorting, grading, processing, and general attention to detail throughout value chains (Parfitt *et al.* 2010).

A troubling consumer-level trend in rural SSA is that food that is thought or known to be contaminated or of poor quality may be diverted to feeding livestock instead. While this may represent a “second best” use of unsafe food, research reveals that animals fed contaminated food often pass the pathogens or toxins through to their milk, which humans often consume, with adverse health consequences (ILRI 2013).

**References**

Allaway, W. (1986). Soil-plant-animal and human interrelationships in trace element nutrition. In W. Mertz (Ed.), *Trace elements in human and animal nutrition.* (pp. 465–488). Orlando, FL: Academic Press.

Barrett, C.B. (1996). Urban Bias in Price Risk: The Geography of Food Price Distributions in Low-Income Economies. *Journal of Development Studies, 32(6):* 830-849.

Barrett, C.B., & Bevis, L.E.M. (2015). The Micronutrient Deficiencies Challenge in African Food Systems. In D.E. Sahn, (Ed.), *The Fight Against Hunger and Malnutrition: The Role of Food, Agriculture, and Targeted Policies.* Oxford: Oxford University Press.

Bechoff, A., Tomlins, K., Dhuique-Mayer, C., Dove, R., & Westby, A. (2011). On-farm evaluation of the impact of drying and storage on the carotenoid content of orange-fleshed sweet potato. *International Journal of Food Science and Technology, 46,* 52–60.

Hell, K., Fandohan, P., Bandyopadhyay, R., Kiewnick, S., Sikora, R., & Cotty, P. J. (2008). Pre- and postharvest management of aflatoxin in maize : An African perspective. In J. F. Leslie, R. Bandyopadhyay, & A. Visconti (Eds.), *Mycotoxins: Detection methods, management, public health and agricultural trade* (pp. 219–229). Wallingford, UK.

Hodges, R. J., Buzby, J. C., & Bennett, B. (2011). Postharvest losses and waste in developed and less developed countries: opportunities to improve resource use. *Journal of Agricultural Science, 149,* 37–45.

ILRI. (2013). "Safer food through risk reduction of mycotoxins within the feed-dairy chain in Kenya." Media Briefing No. 5. Nairobi, Kenya.

Ippolito, A., & Nigro, F. (2000). Impact of preharvest application of biological control agents on postharvest diseases of fresh fruits and vegetables. *Crop Protection, 19,* 715–723.

Kearney, J. (2010). Food consumption trends and drivers. *Philosophical Transactions of the Royal Society, 365,* 2793–2807.

Lauren, J. G., Shrestha, R., Sattar, M.A. & Yadav, R.L. (2001). Legumes and Diversification of the Rice-Wheat Cropping System. In Kataki, P. K.(Ed.), *The Rice-Wheat Cropping System of South Asia: Trends, Constraints, Productivity and Policy.* New York: Food Products Press.

Mason, N., & Myers, R. J. (2013). The effects of the Food Reserve Agency on maize market prices in Zambia. *Agricultural Economics, 44,* 203-216.

Parfitt, J., Barthel, M., & Macnaughton, S. (2010). Food waste within food supply chains: quantification and potential for change to 2050. *Philosophical Transactions of the Royal Society, 365,* 3065–3081.

Pollan, M. (2013). *Cooked: A Natural History of Transformation.* New York: The Penguin Press.

Sitko, N. J., & Kuteya, A. N. (2013). "The Maize Price Spike of 2012/13: Understanding the Paradox of High Prices despite Abundant Supplies." Working Paper No. 81. Indaba Agricultural Policy Research Institute: Lusaka, Zambia.

Stathers, T., Lamboll, R., & Mvumi, B. M. (2013). Postharvest agriculture in changing climates: its importance to African smallholder farmers. *Food Security, 5,* 361–392.

Welch, R. M. & Graham, R.D. (1999). A New Paradigm for World Agriculture: Meeting Human Needs Productive, Sustainable, Nutritious. *Field Crop Research 60 (1–2):* 1–10.

Welch, R. M. (2001). Micronutrients, Agriculture and Nutrition; Linkages for Improved Health and Well Being. In Singh, K., Mori, S. & Welch, R.M. (Eds.), *Perspectives on the Micronutrient Nutrition of Crops.* Jodhpur, India: Scientific Publishers.

Wiatrak, P. J., Wright, D. L., Marois, J. J., & Wilson, D. (2005). Influence of Planting Date on Aflatoxin Accumulation in Bt , non-Bt , and Tropical non-Bt Hybrids. *Agronomy Journal, 97,* 440–445.

World Bank, Natural Resources Institute, & Food and Agriculture Organization. (2011). "Missing Food: The case of postharvest grain losses in Sub-Saharan Africa." Report No. 60371-AFR. Washington, DC.

World Bank. (2009). *World Development Report: Reshaping Economic Geography.* Washington, DC.
